# Determining the power of a 1-sided *z*-test given only the power of the corresponding 2-sided test

**DOI:** 10.1007/s10865-025-00595-6

**Published:** 2025-09-09

**Authors:** Amy Liang, Kristopher J. Preacher, Nathaniel J. Williams, Paul D. Allison, Steven C. Marcus, Sonya K. Sterba

**Affiliations:** 1 Department of Psychology and Human Development, Vanderbilt University, 230 Appleton Place, Nashville, TN 37203‑5721 USA; 2 School of Social Work, Boise State University, Boise, ID USA; 3 Institute for the Study of Behavioral Health and Addiction, Boise State University, 1910 University Drive, Boise, ID 83725‑1940 USA; 4 Statistical Horizons LLC, P.O. Box 282, Ardmore, PA 19003 USA; 5 Penn Center for Mental Health, University of Pennsylvania School of Medicine, 3535 Market Street, Philadelphia, PA 19104 USA; 6 School of Social Policy and Practice, University of Pennsylvania, 3701 Locust Walk, Philadelphia, PA 19104‑6214 USA

**Keywords:** Statistical power, Directional and non-directional hypotheses, Hypothesis testing, Power conversion

## Abstract

Estimating statistical power is essential for designing behavioral medicine studies efficiently and conserving finite resources. Sometimes behavioral medicine researchers are interested in calculating power for 1-sided *z*-tests of individual parameters (e.g., slopes) in complex models such as multilevel structural equation models or multilevel mixture regression models. For such models, calculating power for 1-sided *z*-tests is cumbersome because: (a) online *z*-test power calculator tools are inapplicable, (b) commonly-used power analysis software provides power only for 2-sided *z*-tests and does not allow changing alpha, and (c) published power tables typically provide power results only for 2-sided *z*-tests. Hence, here we introduce straightforward and resource-efficient conversion formulas to estimate the power of 1-sided *z*-tests of individual parameters in any model by using direct power conversions from the corresponding 2-sided tests. We then implement these conversion formulas in accessible R and Excel software. This brief report thus provides behavioral medicine researchers with a convenient and practical solution for power calculation that minimizes the time, financial, and computational resources typically needed for power estimation.

The *power* of a statistical test is the probability of rejecting a false null hypothesis $({H}_{0})$ given that some alternative hypothesis (*H*_A_) is true. Power estimation has been widely recognized as crucial in behavioral medicine research, as it enables researchers to conserve finite research resources, avoid wasting participants’ time, and minimize the chance of failing to detect a true effect (e.g., Davidson et al., 2003). In conducting power analyses, researchers have the option to adopt either non-directional (2-sided) or directional (1-sided) hypotheses.

## Directional hypotheses

Across scientific disciplines, traditional 2-sided tests of non-directional hypotheses are commonly employed, especially in exploratory settings where researchers do not want to hinder discovery of effects in the unexpected direction. An example *non-directional hypothesis* posited by a behavioral medicine researcher is: child cerebral palsy patients treated with myofascial-release techniques plus functional training have *no difference* in gross motor skills compared with those receiving only functional training. However, in reviews of over 490 published applications of structural equation modeling, regression, and ANOVA containing over 2700 stated hypotheses, over 90% of hypotheses stated were actually directional (Cho & Abe, 2012). An example *directional hypothesis* posited by a behavioral medicine researcher is: child cerebral palsy patients treated with myofascial-release techniques plus functional training will have *more improvement* in gross motor skills than those receiving only functional training (Rafat & Srivastav, 2024). When theory is strong enough to suggest an expected direction for an effect, it makes little sense to conduct a 2-sided significance test in which half of the rejection region lies in the opposite, unexpected direction; such a test would be inconsistent with the hypothesis.

Thus, 1-sided tests are a logically consistent choice for evaluating directional hypotheses. 1-sided tests can be useful when there is a reasonable expectation of an outcome occurring in a given direction based on theory or prior knowledge and when a result in the direction opposite of expectation would have little implications for theory or practice (e.g., Hick, 1952; Levitt, 1994; Rice & Gaines, 1994; Ruxton & Neuhäuser, 2010; Wike, 1971). For instance, 1-sided tests are used in placebo-controlled clinical trials to test directional hypotheses that a new drug (e.g., for insomnia) is superior to a placebo—where the new drug would not receive approval if it were less effective than the placebo (e.g., Murphy, 1998). As another example, 1-sided tests are used to test directional hypotheses that product exposure (e.g., BPA [bisphenol-A] in can linings) harms vulnerable groups such as children or pregnant women—necessitating placing a warning label on the product (e.g., Ruxton & Neuhäuser, 2010). No warning label would be affixed if the product had either a neutral or net beneficial effect.

Another reason behavioral medicine researchers might choose directional hypothesis tests is that they are more powerful than their non-directional counterparts and therefore require smaller sample sizes (e.g., Hales, 2024; Hernández et al., 2018; Lawler & Zimmermann, 2021). This is an especially important consideration when the costs of sampling are high, such as in highly pragmatic trials (Ford & Norrie, 2016; Loudon et al., 2015). Consider the simple situation in which a behavioral medicine researcher wishes to conduct a significance test for the effect (slope) of goal-striving (*x*) on chronic pain (*y*) in the context of simple linear regression. Slope estimators quickly approach normality with increasing *n*, justifying the use of an asymptotic *z*-test. Given a slope estimate for the effect of goal-striving on chronic pain of $\hat{b} =.16\left( {SE =.09} \right)$, the test statistic ($z = \hat{b}/SE = 1.78$) would be significant at *α =.05* for the 1-sided test (*p* =.0377) but nonsignificant for the 2-sided test (*p* =.0754). Of course, ethically the decision of whether to use a 1-sided test must be made in advance, not after inspecting 2-sided test results (Burke, 1953; Panter & Sterba, 2011). This can be accomplished during pre-registration of a study or trial (e.g., Bosnjak et al., 2022) wherein the researcher would specify in advance whether hypotheses are non-directional or directional and whether corresponding tests are one-sided or two-sided before analyzing the data (see Lawler & Zimmermann, 2021). This would avoid what Lawler and Zimmerman (2021) call “hypothesis hacking”—formulating or changing a hypothesis after statistical testing to correspond with the testing results.

## Power for 1-sided tests to evaluate directional hypotheses

Thus, directional hypothesis tests may be more consistent with theory and always will be more powerful than non-directional tests. It follows that the power for directional tests can be of interest. Although some online calculators^1^ often can compute power for 1 or 2-sided *z*-tests, such online calculators are designed for simple situations where one knows *n* and wants to compute power (e.g., a *z*-test for testing a single mean against a constant or testing the difference in two means). In those cases, *n* is explicitly included in the *z*-test formula. These online calculators *do not apply* to more complex cases where *z*-tests are used but *n* is not explicitly included in the formula– e.g., tests for slopes in models using maximum likelihood estimation, such as path coefficients in mixture regression models, multilevel structural equation models, multilevel mixture models, etc. that are increasingly of interest in behavioral medicine research (e.g., Baumann et al., 2020; Deng et al., 2022; de Vries McClintock et al., 2014; Kessels et al., 2021; Mun et al., 2016; Romano et al., 2020; St Fleur et al. 2025; Watanabe & Yamauchi, 2016; Wi et al., 2024). For example, in Mun et al. (2016) the slope of chronic pain on goal striving is one parameter embedded in a multilevel structural equation model; thus, power for a one-sided *z*-test of this slope cannot be obtained using such online calculators. In these more complex situations, researchers often (1) turn to power tables from published simulations or (2) conduct Monte Carlo simulations themselves using, for instance, M*plus* (Muthén & Muthén, 1998–2025), simsem (Pornprasertmanit et al., 2021), or WebPower (Zhang & Yuan, 2018)—see Liang and Sterba (in press) for a review of such programs.

Although many behavioral medicine researchers still prefer to use tabled power values instead of running Monte Carlo simulation software programs for power analyses, unfortunately the majority of tabled *z-*test power values provided in published simulation studies (see, e.g., Egbewale et al., 2014; Geminiani et al., 2021; Kaplan & George, 1995; MacKinnon et al., 2002; Preacher et al., 2011; Van Horn et al., 2008) are only for non-directional (2-sided) tests, rather than for directional (1-sided) tests. For researchers who instead opt to use Monte Carlo simulation software programs to compute power for *z*-tests from complex models, unfortunately some frequently-used software programs for this purpose (e.g., M*plus;* Muthén & Muthén, 1998–2025) generate statistical power only for 2-sided, rather than for 1-sided, *z*-tests and do not permit doubling alpha in order to obtain power for 1-sided *z*-tests. Behavioral medicine researchers are thus in need of a method to quickly convert a 2-sided test’s power to a 1-sided test’s power.

To address this limitation, in this brief report we provide behavioral medicine researchers with convenient formulas that facilitate the conversion from power for non-directional (2-sided) *z*-tests to power for directional (1-sided) *z-*tests, when only non-directional (2-sided) power values are available. Our conversion formulas are applicable under the same outcome-normality assumptions invoked when determining power for *z*-tests using published power tables mentioned earlier or using typical implementations of Monte Carlo power analyses (Liang & Sterba, in press), that don’t explicitly involve generating nonnormal data. We also implement these conversion formulas using widely-used R and Excel software. Readers wishing to bypass the derivation can skip to the software implementation section.

## Power of a 1-sided *z*-test

Please refer to Table 1 for a comprehensive reference list of all symbols used in equations, and their definitions. Although the following presentation assumes the alternative hypothesis $H_{1}:\theta > \theta_{0}$—i.e., that the proposed effect *θ* is greater than its null-hypothesized value $\theta_{0}$—analogous logic applies when $\theta < \theta_{0}$ such that, if indeed $H_{1}:\theta < \theta_{0}$, our final formula (i.e., Eq. 15) still holds, as we describe later.

**Table 1:** Symbols and their meanings

Symbols	Meaning
*Φ*	Standard normal cdf,^a^ which converts *z*-scores into *p*-values
$\Phi^{ - 1}$	Inverse standard normal cdf, which converts *p*-values into *z*-scores
*α*	Nominal Type I error rate
*n*	Total sample size
*θ*	Proposed/theorized effect^b^
$\hat{\theta }$	Estimated/predicted effect
$\theta_{0}$	Hypothesized effect under $H_{0}$
$\theta_{c1}$	Critical value under ${H}_{0}$ for the 1-sided test
$\theta_{c2 + }$	Right-side critical value under ${H}_{0}$ for the 2-sided test
$\theta_{c2 - }$	Left-side critical value under ${H}_{0}$ for the 2-sided test
*σ*	Standard error of $\hat{\theta }$
$z_{N1}$	*z*-score associated with $\theta_{c1}$ under ${H}_{0}$ for the 1-sided test
$z_{N2 + }$	*z*-score associated with $\theta_{c2 + }$ under ${H}_{0}$ for the 2-sided test
$z_{N2 - }$	*z*-score associated with $\theta_{c2 - }$ under ${H}_{0}$ for the 2-sided test
$z_{A1}$	*z*-score associated with $\theta_{c1}$ under ${H}_{1}$ for the 1-sided test
$z_{A2 + }$	*z*-score associated with $\theta_{c2 + }$ under ${H}_{1}$ for the 2-sided test
$z_{A2 - }$	*z*-score associated with $\theta_{c2 - }$ under ${H}_{1}$ for the 2-sided test
$\pi_{1}$	Power for the 1-sided test
$\pi_{2}$	Power for the 2-sided test

^a^cdf, cumulative distribution function

^b^The term “effect” is used here to refer to any parameter that reflects a hypothesis of interest. In this short report, our discussion pertains to testing parameters for which *z*-tests are appropriate (e.g., slopes, intercepts, and mean differences)

**Fig. 1:**
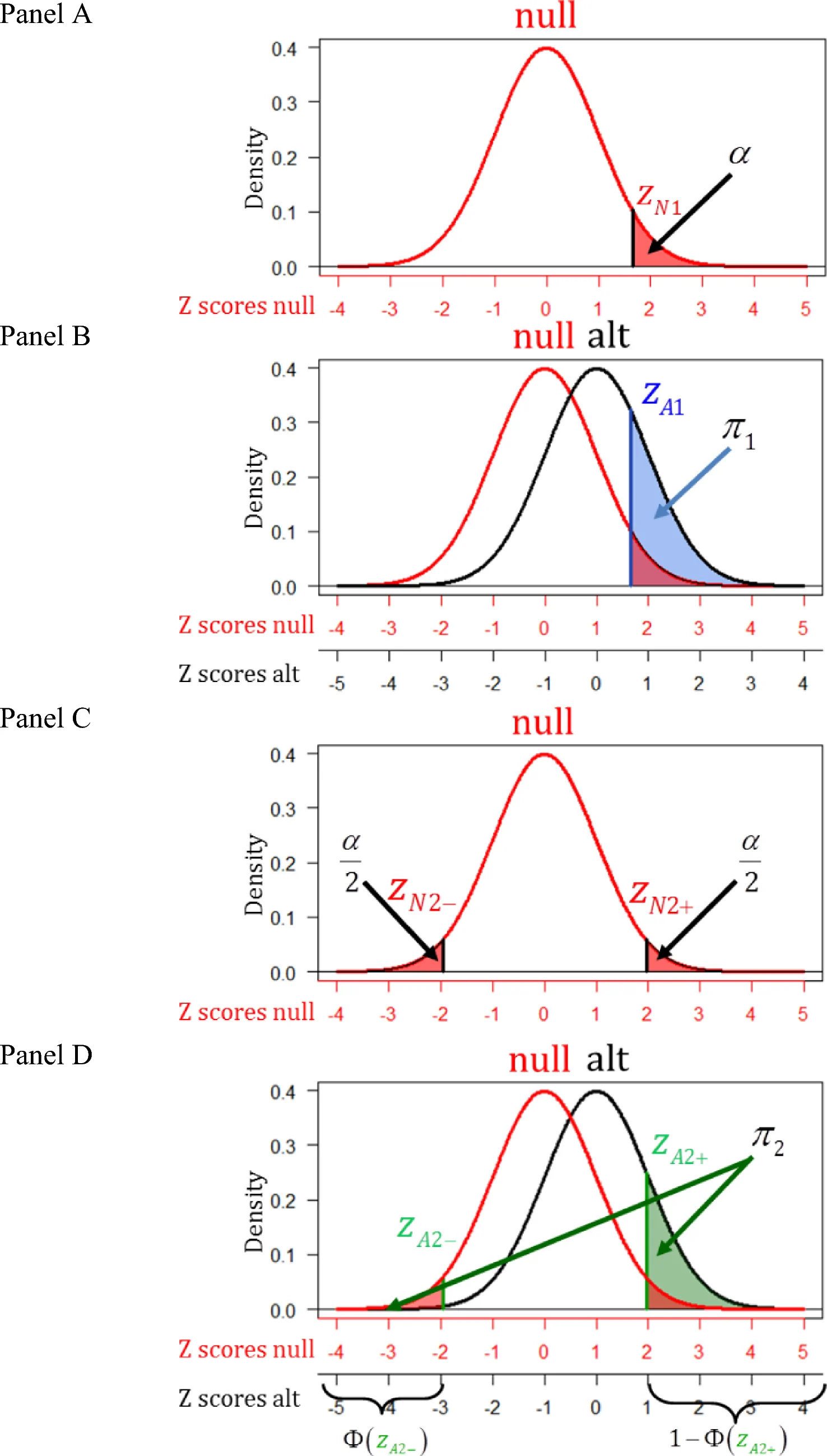
Diagrams to visualize 1-sided power ($\pi_{1}$) and 2-sided power ($\pi_{2}$) of a *z*-test, where *H*_1_: $\theta > \theta_{0}$

Under the alternative hypothesis $H_{1}:\theta > \theta_{0}$, in a 1-sided *z*-test, the critical value of *z* under $H_{0}$ at a specified significance level, denoted $z_{N1}$, can be visualized in Fig. 1 Panel A and is defined as:

1 $$ z_{N1} = \Phi^{ - 1} \left( {1 - \alpha } \right) = \frac{{\theta_{c1} - \theta_{0} }}{\sigma } $$

where $\theta_{c1}$ is the corresponding critical value of the parameter being tested. Thus,

2 $$ \theta_{c1} = \sigma \Phi^{ - 1} \left( {1 - \alpha } \right) + \theta_{0} $$

The value of *z* for the same $\theta_{c1}$ under $H_{1}$ (i.e., that *θ* is the proposed effect) is denoted $z_{A1}$. This $z_{A1}$ is represented in Fig. 1 Panel B and is expressed as

3 $$ z_{A1} = \frac{{\theta_{c1} - \theta }}{\sigma } = \frac{{\sigma \Phi^{ - 1} \left( {1 - \alpha } \right) + \theta_{0} - \theta }}{\sigma } = \Phi^{ - 1} \left( {1 - \alpha } \right) + \frac{{\theta_{0} }}{\sigma } - \frac{\theta }{\sigma } $$

Thus, a quantity that will be used later in deriving the conversion formula is

4 $$ \frac{\theta }{\sigma } = \Phi^{ - 1} \left( {1 - \alpha } \right) + \frac{{\theta_{0} }}{\sigma } - z_{A1} $$

The probability of rejecting $H_{0}$ when $H_{1}$ is true—i.e., power for the 1-sided *z*-test—is denoted $\pi_{1}$:

5 $$ \pi_{1} = 1 - \beta_{1} = 1 - \Phi \left( {z_{A1} } \right) $$

$\pi_{1}$ can be visualized in Fig. 1 Panel B.

## Power of a 2-sided *z*-test

Unlike 1-sided tests, a 2-sided *z*-test involves both left-side ($\theta_{c2 - }$) and right-side ($\theta_{c2 + }$) critical values of the parameter being tested, under $H_{0}$. The right-side critical value of *z* under $H_{0}$, denoted $z_{N2 + }$ is depicted in Fig. 1 Panel C and is defined as:

6 $$ z_{N2 + } = \Phi^{ - 1} \left( {1 - \frac{\alpha }{2}} \right) = \frac{{\theta_{c2 + } - \theta_{0} }}{\sigma } $$

Thus, the right-side critical value of the parameter being tested, $\theta_{c2 + }$, is:

7 $$ \theta_{c2 + } = \sigma \Phi^{ - 1} \left( {1 - \frac{\alpha }{2}} \right) + \theta_{0} $$

The value of *z* for the same $\theta_{c2 + }$ under $H_{1}$, denoted $z_{A2 + }$, is illustrated in Fig. 1 Panel D and can be expressed as:

8 $$ \begin{aligned} & z_{{A2 + }} = \frac{{\theta _{{c2 + }} - \theta }}{\sigma } = \frac{{\sigma \Phi ^{{ - 1}} \left( {1 - \frac{\alpha }{2}} \right) + \theta _{0} - \theta }}{\sigma } \\ & \quad = \Phi ^{{ - 1}} \left( {1 - \frac{\alpha }{2}} \right) + \frac{{\theta _{0} }}{\sigma } - \frac{\theta }{\sigma } \\ \end{aligned} $$

Thus, a quantity to be used later when deriving the conversion formula is:

9 $$ \frac{\theta }{\sigma } = \Phi^{ - 1} \left( {1 - \frac{\alpha }{2}} \right) + \frac{{\theta_{0} }}{\sigma } - z_{A2 + } $$

The power for the 2-sided *z*-test, denoted $\pi_{2}$, is thus given in Eq. (10) and is illustrated in Fig. 1 Panel D:

10 $$ \pi_{2} = 1 - \beta_{2} = 1 - \Phi \left( {z_{A2 + } } \right) + \Phi \left( {z_{A2 - } } \right) $$

where $z_{A2 - }$ is the value of *z* corresponding to $\theta_{c2 - }$ under $H_{1}$. Under the assumption of normality necessary for applying *z*-tests, and when^2^$H_{1}:\theta > \theta_{0}$, the $\Phi \left( {z_{A2 - } } \right)$ becomes negligible (as demonstrated later, and as illustrated in Fig. 1 Panel D), adding only trivially to power.

## Derivation of a formula converting power for a 2-sided *z*-test to power for a 1-sided *z*-test

Setting both of the above expressions for $\frac{\theta }{\sigma }$, in Eqs. (4) and (9), to be equal, we can then derive the *z*-score of the critical value $\theta_{c1}$ under ${H}_{1}$, denoted *z*_*A*1_, in terms of *z*_*A*2+_:

11 $$ \begin{aligned} & \Phi^{ - 1} \left( {1 - \frac{\alpha }{2}} \right) + \frac{{\theta_{0} }}{\sigma } - z_{A2 + } = \Phi^{ - 1} \left( {1 - \alpha } \right) + \frac{{\theta_{0} }}{\sigma } - z_{A1} \\ & z_{A1} = \Phi^{ - 1} \left( {1 - \alpha } \right) - \Phi^{ - 1} \left( {1 - \frac{\alpha }{2}} \right) + z_{A2 + } \\ \end{aligned} $$

Now we can take our earlier Eq. (5) expression for 1-sided power $\pi_{1}$ and substitute Eq. (11) for *z*_*A*1_:

12 $$ \begin{aligned} & \pi_{1} = 1 - \beta_{1} = 1 - \Phi \left( {z_{A1} } \right) \\ & \quad = 1 - \Phi \left( {\Phi^{ - 1} \left( {1 - \alpha } \right) - \Phi^{ - 1} \left( {1 - \frac{\alpha }{2}} \right) + z_{A2 + } } \right) \\ \end{aligned} $$

But we would like $\pi_{1}$ to be expressed in terms of two-sided power $\pi_{2}$ (rather than in terms of $z_{A2 + }$), so we solve for $z_{A2 + }$ using an approximation further discussed in the next section:

13 $$ \begin{aligned} & \pi _{2} = 1 - \Phi \left( {z_{{A2 + }} } \right) + \Phi \left( {z_{{A2 - }} } \right) \\ & \Phi \left( {z_{{A2 + }} } \right) = 1 - \pi _{2} + \underbrace {{\Phi \left( {z_{{A2 - }} } \right)}}_{{{\text{essentially 0}}}} \\ & \Phi \left( {z_{{A2 + }} } \right) \approx 1 - \pi _{2} \\ & \Phi ^{{ - 1}} \left( {\Phi \left( {z_{{A2 + }} } \right)} \right) \approx \Phi ^{{ - 1}} \left( {1 - \pi _{2} } \right) \\ & z_{{A2 + }} \approx \Phi ^{{ - 1}} \left( {1 - \pi _{2} } \right) \\ \end{aligned} $$

Finally, substituting Eq. (13) into Eq. (12) yields the following approximation formula for converting power for a 2-tailed *z*-test, $\pi_{2}$, to power for the 1-tailed *z*-test, $\pi_{1}$:

14 $$ \pi_{1} \approx 1 - \Phi \left( {\Phi^{ - 1} \left( {1 - \alpha } \right) - \Phi^{ - 1} \left( {1 - \frac{\alpha }{2}} \right) + \Phi^{ - 1} \left( {1 - \pi_{2} } \right)} \right) $$

A mathematically equivalent, but slightly briefer, formula is given in Eq. (15):

15 $$ \pi_{1} \approx \Phi \left( {\Phi^{ - 1} \left( \alpha \right) - \Phi^{ - 1} \left( {\frac{\alpha }{2}} \right) + \Phi^{ - 1} \left( {\pi_{2} } \right)} \right) $$

In other words, for a given significance level *α* and a power estimate $\pi_{2}$ from a 2-sided *z*-test, we can use Eq. (15) to approximate the power of the corresponding 1-sided *z*-test $\pi_{1}$.

## Adequacy of the approximation used in the conversion formula

**Fig. 2:**
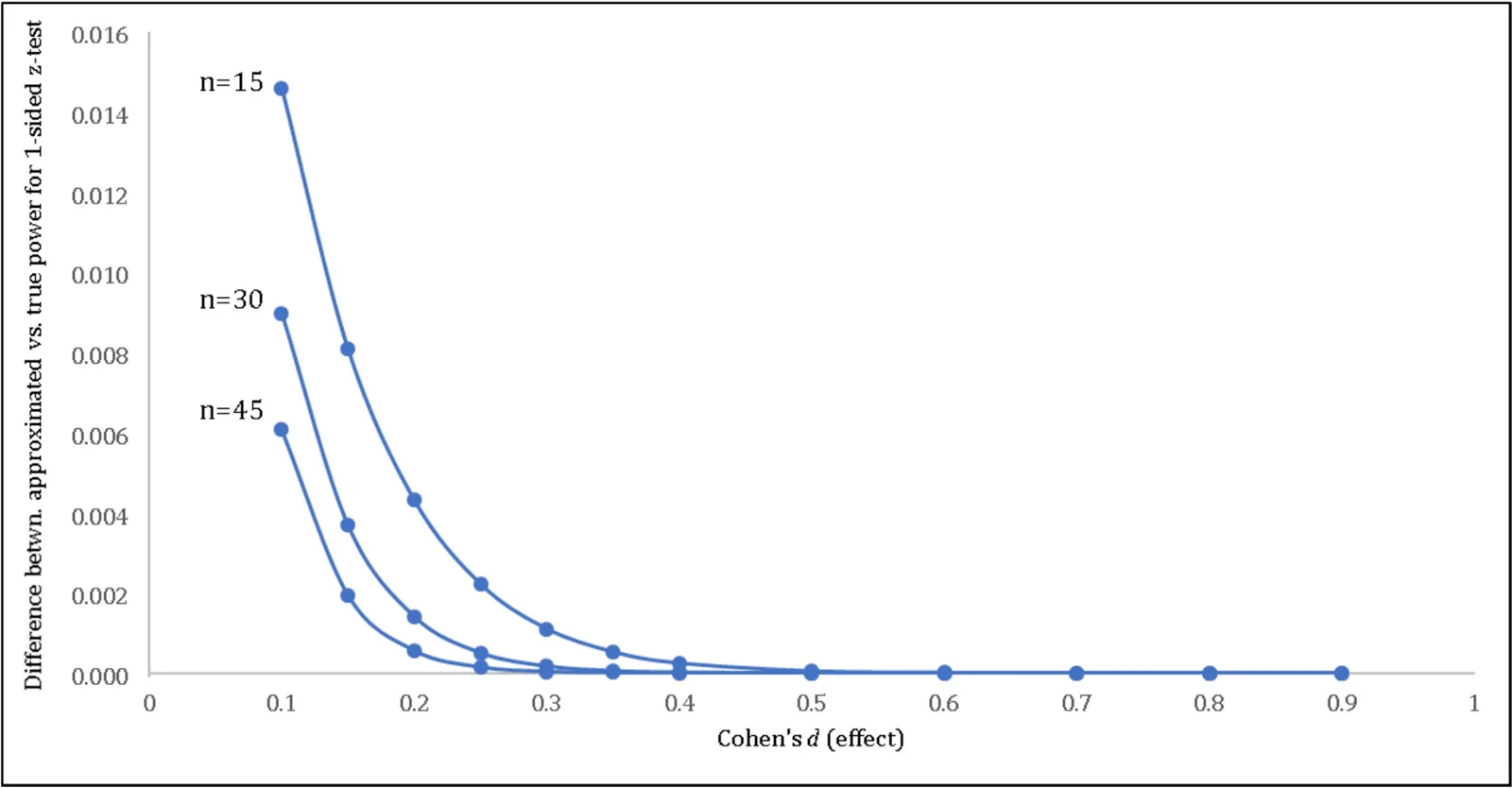
Relationship between approximated (using conversion formula) vs. true power for a 1-sided *z*-test, across sample sizes and target effects at *α* =.05

As a consequence of ignoring the trivial contribution of $\Phi \left( {z_{A2 - } } \right)$ (i.e., the tiny probability under the opposing side of the alternative distribution from the 2-sided test) in Eq. (13), our estimate of $\Phi \left( {z_{A2 + } } \right)$ and $z_{A2 + }$ in Eq. (13) will be negligibly decreased. This implies that, in the approximation formula for 1-tailed power $\pi_{1}$ (Eq. (14)), the argument to the outer *Φ* will be negligibly decreased, so our estimate of $\pi_{1}$ will be negligibly inflated, to a degree we here show is trivial—under the conventional normality assumption necessary for the use of *z*-tests. Figure 2 shows that, assuming normality, this inflation is inconsequential as it alters estimates for 1-tailed power $\pi_{1}$ only trivially (inflation of ~.01, on the vertical axis) for small target effects on the horizontal axis (such as Cohen’s *d* of .10) and—for more typically-conjectured effect sizes—alters power estimates by  ~.00, on the vertical axis, for modest to large effects on the horizontal axis (such as Cohen’s *d* ≥.20) across sample size (*n*), at *α* =.05. One may consider how Fig. 2 would look for a different *α*. Values of α >.05 are uncommonly used and *α* <.05 would result in even smaller $\Phi \left( {z_{A2 - } } \right)$ and thus even smaller amounts of inflation. If an unusually large *α* =.10 were used—which is uncommon—the inflation would still be  ~.00 for Cohen’s *d* ≥.25 but for Cohen’s *d* =.10 would range from.029 for *n* = 15 to.013 for *n* = 45.

What if the conventional normality assumption that underlies the use of *z*-testing is markedly violated? Then it may be useful to consider alternatives to *z*-tests entirely, avoid consulting *z*-test power tables generated under normality assumptions, and avoid converting 2-tailed power values—obtained assuming normality—to 1-tailed power values that also assume normality. Nonnormality stemming from multimodality, outliers, skewness, etc. each could have different impacts on accuracy of power calculations, and such patterns could be investigated via a simulation study like the following. First, a researcher could simulate hundreds or thousands of samples from a population consistent with the null hypothesis, with the anticipated kind/degree of nonnormality in the outcome. Each simulated sample could be used to produce a test statistic—cumulatively forming an empirical null distribution. For, say, *α* =.05, a right-side empirical critical value $\hat{z}_{N1}^{ \bullet }$—where the superscript *∙* denotes that a normal distribution is no longer assumed—can be determined by identifying the test statistic value at the 95th percentile of this empirical null distribution. Second, the researcher could simulate thousands of samples consistent with the alternative hypothesis—again with the anticipated kind/degree of outcome nonnormality, to produce an empirical distribution of test statistics consistent with that alternative hypothesis. Third, the researcher then could calculate the area beyond $\hat{z}_{N1}^{ \bullet }$ under that empirical alternative distribution to empirically determine 1-tailed power without having to assume normality (see also Table 4 Approach 3 from Liang & Sterba, in press). Fourth, the latter result could then be compared to 1-tailed power obtained using our conversion formula under conventional normality assumptions—in order to ascertain the impact of nonnormality on power estimates obtained using our conversion formula.

## Software implementation of the conversion formula and illustrative example

The power conversion described in this report can be accomplished using simple commands in widely available software, such as R and Excel, as shown here.

*Implementation in Excel*. For example, in an empty Microsoft Excel spreadsheet, one could insert (i.e., enter) *α* in cell B1 of the spreadsheet, and insert (i.e., enter) the power of the 2-sided test ($\pi_{2}$) in cell B2 of the spreadsheet. In a third cell of the same Excel spreadsheet the researcher could then copy and paste the following code—which refers to cell B1 (where the researcher has already inserted their desired *α*) and to cell B2 (where the researcher has already inserted their $\pi_{2}$)—in order to obtain the approximate power of the 1-sided *z*-test ($\pi_{1}$):

=NORM.S.DIST(NORM.S.INV(B1)-NORM.S.INV(B1/2)+NORM.S.INV(B2),TRUE)

Simplifying further, if we can assume *α =.05*, the right-side critical value of *z* for a 2-sided *z-*test is 1.95996, and the critical value for a 1-sided *z*-test is 1.64485, then the researcher could instead paste into the same Excel spreadsheet the following simplified equation code:

=NORM.S.DIST(0.31511+NORM.S.INV(B2),TRUE)

which requires only $\pi_{2}$ as input (where again $\pi_{2}$ is 2-tailed power that the researcher previously inserted into cell B2 of the same Excel spreadsheet).

*Implementation in R*. Similarly, $\pi_{1}$ can be readily computed in R software by first defining alpha as the significance level (e.g., if the desired level were.05 this could be done using R code: alpha =.05) and then defining two.tail.pi in R as the input $\pi_{2}$, i.e., the 2-tailed power. Next, the below code could be copied and pasted into R:

pnorm(qnorm(alpha)-qnorm(alpha/2)+qnorm(two.tail.pi))

as will be demonstrated with a screen shot in the next section. Importantly, the approximation in Eq. 15 and the Excel and R implementations work both when H_1_: $\theta > \theta_{0}$ *or* when H_1_: $\theta < \theta_{0}$.

*Empirical example*. Next we consider an illustrative application of the conversion formula. Recently, Geminiani et al. (2021) published a Monte Carlo simulation that provided a power table for 2-sided *z*-tests to detect across-group differences in factor loadings in a multilevel factor mixture model. In one design cell, an absolute difference between loadings of  ~.30 was detectable with.69 power at *α =.05* using a 2-tailed *z*-test. Yet, a future behavioral medicine researcher designing a study may want to know 1-tailed power under this condition. Because the published table provided only 2-tailed power, the future behavioral medicine researcher would be faced with running a computationally-intensive simulation to determine 1-tailed power for this test. Instead, in this situation 1-tailed power $\pi_{1}$ for the *z*-test can be quickly calculated by that researcher to be.79 by implementing our conversion formula using the above R or Excel code. In the below screen shot, the three lines of R input are followed by the output, on the fourth line.

**Figure:**
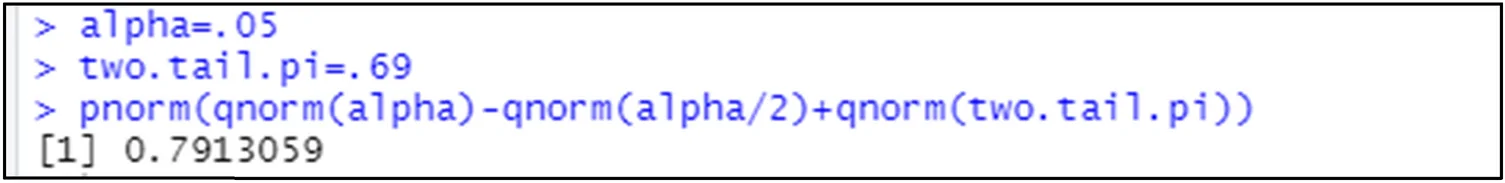

Or, the same 1-tailed power for the *z*-test can be readily calculated using our conversion formula implemented in Excel as described above:

**Figure:**
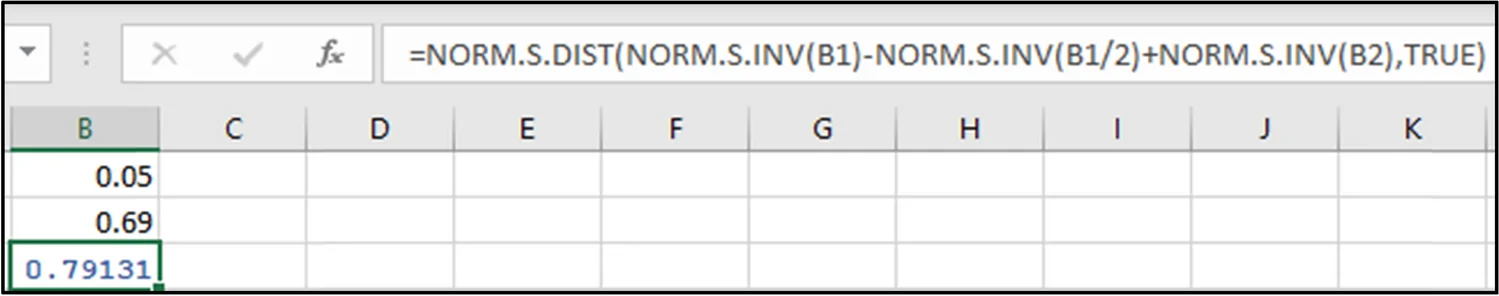

## Discussion

In conclusion, power of 2-sided *z-*tests is readily available from published power tables or Monte Carlo power analysis software default output; however, this often is not the case for power of 1-sided *z-*tests. For situations where theory and study goals lead to an interest in power for 1-tailed tests (e.g., Williams et al., 2022), this study introduces a direct and easy method for behavioral medicine researchers to approximate power for 1-sided *z*-tests given an estimate of the power of a 2-sided test.

Our approximation method relies on the same conventional normality assumptions underlying *z*-tests. As mentioned earlier, our approximation method can be less accurate for the combination of very low *n* ≤ 15, very small effect size <.10, and unusually large *α* ≥.10; sample sizes this small combined with effect sizes this small and *α* levels this large are less common in practice.

Our approximation method can allow behavioral medicine researchers to efficiently evaluate directional hypothesis tests without incurring costs in terms of time, money, and computational resources. Although this study focuses on power computation in the context of *z*-tests, future research could investigate the feasibility of this strategy for other tests.

## Data Availability

N/A.

## Footnotes

1 Examples of such online calculators include: https://www.statskingdom.com/32test_power_t_z.html and https://mathcracker.com/power-calculator-testing-one-mean.

2 Our derived conversion formula in Eq. (15) holds whether $H_{1}:\theta > \theta_{0}$ or $H_{1}:\theta < \theta_{0}$, as described later; for simplicity, our derivations are presented under the assumption $H_{1}:\theta > \theta_{0}$.

## Code availability

Included in manuscript.
